# Oncostatin M suppresses metastasis of lung adenocarcinoma by inhibiting SLUG expression through coordination of STATs and PIASs signalings

**DOI:** 10.18632/oncotarget.10939

**Published:** 2016-07-29

**Authors:** Chih-Ming Pan, Mong-Lien Wang, Shih-Hwa Chiou, Hsiao-Yun Chen, Cheng-Wen Wu

**Affiliations:** ^1^ Institute of Biochemistry and Molecular Biology, National Yang-Ming University, Taipei, Taiwan; ^2^ Institute of Pharmacology, National Yang-Ming University, Taipei, Taiwan; ^3^ Institute of Clinical Medicine, National Yang-Ming University, Taipei, Taiwan; ^4^ Department of Medical Research and Education, Taipei Veterans General Hospital, Taipei, Taiwan; ^5^ Institute of Biomedical Science, Academia Sinica, Taipei, Taiwan

**Keywords:** oncostatin M, SLUG, STAT1, PIAS4, metastasis

## Abstract

Oncostatin M (OSM) is linked with multiple biological responses including growth and differentiation. Previous reports showed inhibitory effects of OSM in tumor progression while others showed promoting effects. The dual role of OSM in the development of various cancers is still unclear. We previously described OSM-mediated SLUG suppression, leading to repressed metastasis of lung adenocarcinoma (LAC) cells. However, the underlying mechanism remains elusive. Here, we showed that OSM suppresses SLUG express in LAC cells through a STAT1-dependent transcriptional inhibition. Knockdown of STAT1 reversed the OSM-suppressed SLUG expression and rescued the OSM-mediated inhibition of cell proliferation, migration, and invasion *in vitro*, as well as pulmonary metastasis *in vivo*. STAT1 suppressed SLUG transcription through binding to its promoter region in response to OSM. Furthermore, PIAS4, a co-repressor of STAT, and HDAC1 were able to bind to STAT1 on SLUG promoter region, resulting in reduced H3K9 acetylation and suppressed SLUG expression upon OSM treatment. In contrast, PIAS3 bound to activated STAT3, another effector of OSM, in response to OSM and blocked the binding of STAT3 to SLUG promoter region, preventing STAT3-dependent activation of SLUG transcription. Our findings suggested that OSM suppresses SLUG expression and tumor metastasis of LAC through inducing the inhibitory effect of the STAT1-dependent pathway and suppressing the activating effect of STAT3-dependent signaling. These results can serve as a scientific basis for the potential therapeutic intervention of OSM in cancer cells.

## INTRODUCTION

Lung cancer is one of the most common causes of cancer-related mortality [1]. Above all, lung adenocarcinoma (LAC) is the most frequent histologic type with a high metastatic incidence of lung cancer. The poor prognosis of LAC may attribute to its highly metastatic potential and frequent recurrence incidence [2]. The epithelial-mesenchymal transition (EMT) was found to be involved in carcinoma metastasis, resistance to apoptosis, and properties of cancer stemness [3, 4]. The process of EMT is controlled by various transcription factors which are activated by intrinsic or extrinsic stimuli and regulated the phenotypic and functional changes of cancer cells. In lung cancer, SLUG is a predominant EMT regulator [5]. Elevated expression of SLUG is associated with cancer invasion and poor outcome of patients with LAC [6]. Targeting the EMT pathway or inducing the mesenchymal–epithelial transition (MET) has been reported to suppress lung cancer progression and metastasis [7]. For LAC, targeting SLUG to suppress its expression has been demonstrated to inhibit LAC tumor metastasis in the mouse model [8].

Oncostatin M (OSM) is a multi-functional cytokine of the interleukin-6 (IL-6) family [9]. Ligand binding of OSM to its receptor activates several intracellular signaling proteins, mainly Janus kinase (JAK)-Signal Transducer and Activator of Transcription (STAT) pathway [9, 10]. Activation of these downstream pathways regulates cell proliferation, differentiation, survival and cellular function of hepatocytes [11]. We previously demonstrated that mesenchymal stem cells (MSCs) secrete OSM which effectively inhibited the metastasis and growth of LAC cells [12]. OSM induced the MET pathway, as evidenced by the reduction of several EMT-related markers, including SLUG. However, the underlying mechanism of how OSM mediates the suppression of SLUG is still unknown. Moreover, the OSM is well characterized to activate both STAT1 and STAT3 signalings. According to previous studies, STAT3 has been shown to promote tumor progression, whereas STAT1 tend to suppress it [13, 14]. The coordination between STAT1 and STAT3 upon OSM stimulation is still waiting to be investigated.

In this study, we investigated the underlying mechanism and the involvement of STAT1/STAT3 signaling in the OSM-dependent suppression of SLUG in LAC. Our results revealed how OSM suppressed SLUG expression at the transcription level through coordinating the STAT1 and STAT3 regulatory network.

## RESULTS

### OSM inhibited SLUG expression and cell migration in LAC cells with both STAT1 and STAT3 activations

**Figure 1:**
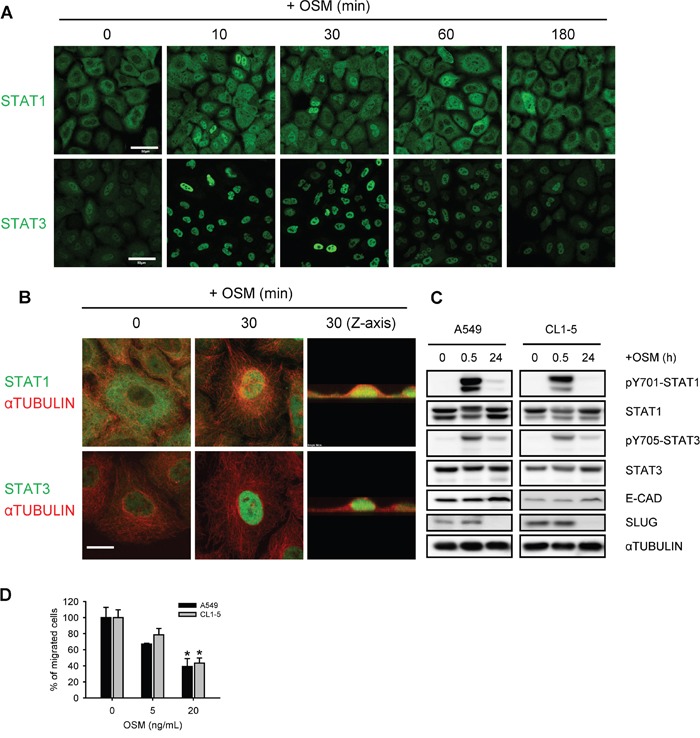
OSM induced the tyrosine phosphorylation and nuclear translocation of STAT1 and STAT3 **A, B.** A549 cells treated with 20 ng/mL OSM for the indicated durations were subjected to immunofluorescent staining of STAT1, STAT3 (green) and αTUBULIN (red), and observed by confocal microscope. Scale bar = 50 μm in (A), 10 μm in (B). **C.** A549 and CL1-5 cells were subjected to Western blotting analysis after 0.5 and 24 hours of incubation in 20 ng/mL OSM. pY701-STAT1 and pY705-STAT3 indicated the phosphorylated STAT1 and STAT3, respectively. **D.** A549 and CL1-5 cells were subjected to the migration assay in the presence of 5 or 20 ng/mL OSM. Sterile water was used as control vehicle for non-treated cells. The results are presented as the percentage of migrated cells treated with OSM relative to non-treated control.

OSM was well-known to activate the JAK-STAT1/3 pathway in various cell types [10]. We firstly tested the activation status of STAT1 and STAT3, upon OSM stimulation in LAC cells. Immunofluorescent images showed that STAT1 and STAT3 were phosphorylated and translocated to the nucleus after 10 minutes of OSM treatment in A549 LAC cell lines (Figure 1A and 1B; Supplementary Figure S1A). The phosphorylation of STAT1 and STAT3 lasted for approximately 60 minutes. Similarly, Western blot analysis of nuclear and non-nuclear fractionated lysates from time-course OSM-treated A549 cells showed a rapid increase of phosphorylation and nuclear translocation of STAT1 and STAT3 (Supplementary Figure S1B). It has to notice that though both STAT1 and STAT3 seemed to be activated, the protein levels of SLUG were decreased 24 hours after OSM treatment (Figure 1C; Supplementary Figure S1B), accompanied with elevated E-cadherin protein level (Figure 1C). In line with the decreased SLUG and increased E-cadherin, OSM decreased the migration of LAC cells in a dose-dependent manner (Figure 1D). These data indicated OSM rapidly induced the phosphorylation and nuclear translocation of both STAT1 and STAT3, and yet decreased SLUG expression and cell migration in LAC cells.

### STAT1 mediated OSM-induced suppression of SLUG expression, migration, proliferation, and experimental metastasis *in vivo*

**Figure 2:**
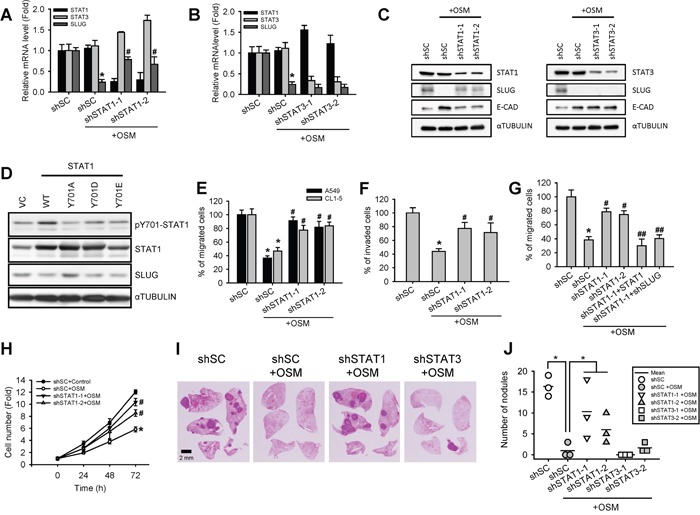
Knockdown of STAT1 increased SLUG level and enhanced cell motility and tumor metastasis **A, B.** A549 cells with stable knockdown of STAT1 (shSTAT1-1 and -2) and STAT3 (shSTAT3-1 and -2) as well as cells with scrambled shRNA control (shSC) were treated with or without OSM (20 ng/mL) for 24 hours and subjected to quantitative real-time PCR for analyzing the mRNA expression level of STAT1, STAT3 and SLUG. The data are presented as relative fold changes to the cells transfected with shSC. An asterisk (*) indicated the statistically significant difference between experimental group and shSC cells without OSM; a hash mark (#) indicated the statistically significant difference between experimental group and shSC with OSM. **C.** A549 cells were treated with or without recombinant OSM (20 ng/mL) for 24 hours before subjected to a Western blotting analysis. **D.** Western blotting analysis of A549 cells stably overexpressing wild-type (WT) or mutated (Y701A, Y701D, and Y701E) STAT1. **E.** A549 and CL1-5 cells stably transfected with shSC or shSTAT1 were treated with or without OSM (20 ng/mL) for 24 hours, and then subjected to a Transwell migration assay. The results are presented as the percentage of migrated shSTAT1 cells relative to the number of migrated shSC cells. **F.** A549 cells stably transfected with shSC or shSTAT1 were treated with or without OSM (20 ng/mL), and then subjected to a Transwell invasion assay. The results are presented as the percentage of invaded shSTAT1 cells relative to the number of invaded shSC cells. **G.** A549 cells stably transfected with shSC, shSTAT1 (shSTAT1-1 and -2), as well as additional transfected with STAT1-overexpresing or SLUG-knockdown plasmid on top of shSTAT1 (shSTAT1-1+STAT1 and shSTAT1+shSLUG, respectively) were treated with or without OSM (20 ng/mL) for 24 hours, and then subjected to a Transwell migration assay. The number of migrated cells were calculated and presented as a relative percentage of the number of migrated shSC cells without OSM. An asterisk (*) indicated the statistically significant difference between experimental group and shSC cells without OSM; a hash mark (#) indicated the statistically significant difference between experimental group and shSC with OSM; The double hash mark (##) indicated the statistically significant difference between experimental group and shSTAT1-1 cells with OSM. **H.** A549 cells were subjected to an alamarBlue cell viability assay in the presence of OSM (20 ng/mL) or control (sterile water) for 3 days. The proliferation curves are shown as the fold changes in cell number. **I.** Immunocompromised mice were transplanted through tail veins with shSC, shSTAT1 and shSTAT3 A549 cells pretreated with or without OSM (20 ng/mL). Mice were sacrificed 6 weeks after transplantation. Tumor formation in lung and histochemical staining of the A549 xenograft tumor sections were photographed. **J.** The numbers of metastatic nodules in the lung were counted and plotted.

To further investigate the downstream effectors of OSM-dependent suppression of SLUG, we knocked down STAT1 (shSTAT1) and STAT3 (shSTAT3), the well-known downstream transcription factors activated by OSM, in A549 and CL1-5 LAC cell lines using lentiviral expressed short hairpin RNAs (shRNA) (Supplementary Figure S1C to E). Knockdown of STAT1 induced a mesenchymal-like morphology in both cell lines (Supplementary Figure S1F). In OSM-treated cells, shSTAT1 reversed the OSM-suppressed SLUG expression, resulting in a higher SLUG protein in comparison to scrambled shRNA control (shSC; Figure 2A and 2C). On the other hand, shSTAT3 decreased both mRNA and protein levels of SLUG and induced an epithelial-like morphology with more cell-cell contact, compared with shSC cells (Figure 2B and 2C; Supplementary Figure S1F). These results suggested that STAT1 was the effector of OSM-dependent reduction of SLUG mRNA and protein expression, and mediated the MET of LAC cells. On the contrary, STAT3 may maintain or activate SLUG expression. To evaluate the importance of STAT1 phosphorylation in its role to mediate SLUG suppression, we established two phosphomimetic (Tyr701 to Asp or Glu) and one non-phosphorylated (Tyr701 to Ala) amino acid substitutions of STAT1 and overexpressed these mutated as well as wild-type STAT1 in A549 cells. Western blot assay showed that the SLUG protein was reduced by overexpressed wild-type STAT1 (Figure 2D); the STAT1-Y701D and STAT1-Y701E phosphomimetic mutation even further reduced SLUG levels, comparing to wild-type STAT1. On the contrary, the non-phosphorylated STAT1-Y701A lost its ability to suppress SLUG. These data further proved that the phosphorylation on tyrosine 701 of STAT1 protein is essential for suppressing SLUG level.

Migration and invasion are critical properties for cancer cells to initiate metastasis [4]. To clarify the involvement of STAT1 in OSM-mediated LAC motility, a Transwell assay was carried out to further assess cell motility. In line with our previous study [12], reduced migration and invasion of LAC cells were observed when cells treated with OSM. Knockdown of STAT1 was able to reverse OSM-induced suppression of cell motility (Figure 2E and 2F). Moreover, over-expression of STAT1 or knockdown of SLUG in shSTAT1 cells abrogated the shSTAT1-mediated reverse and resulted in a migration ability similar to OSM-treated control cells (Figure 2G). Knockdown of STAT1 (shSTAT1) also diminished OSM-induced suppression of cell proliferation (Figure 2H). To evaluate the anti-metastatic effect of STAT1 *in vivo*, we conducted a tail vein injection of A549 cells with shSTAT1, shSTAT3 and shSC pretreated with PBS or OSM in immunocompromised mice. The tumor nodules in the lungs were shown in Figure 2I. The data clearly showed that OSM reduced the number of metastatic tumor nodules in lungs, whereas knockdown of STAT1 abrogated the inhibitory effect (Figure 2J). On the other hand, knockdown of STAT3 resulted in reduced number of nodules to a level similar to OSM-treated control. Taken together, these data showed that STAT1 reduced SLUG expression and mediated the OSM-dependent suppression of cell motility *in vitro* and tumor metastasis *in vivo*, while STAT3 did not interfere with this effect even though it was phosphorylated.

### STAT1 decreased SLUG transcription through direct promoter binding

**Figure 3:**
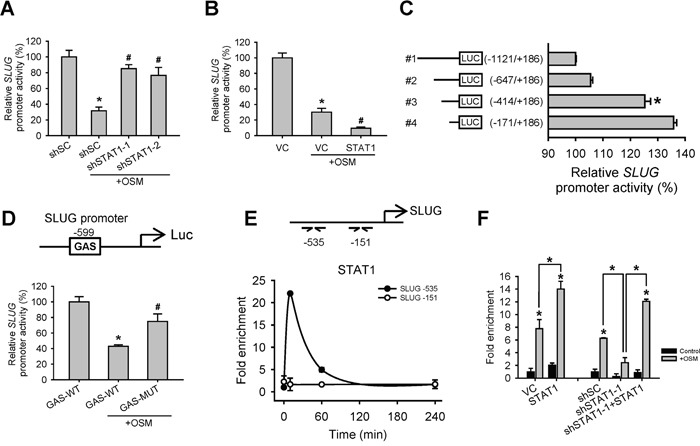
OSM induced STAT1 binding to SLUG promoter region to decrease SLUG promoter activity **A.** A549 cells with stable knockdown of scramble control (shSC) or STAT1 (shSTAT1-1 and -2) were transfected with SLUG promoter (−1121 to +186)-driven luciferase-expressing reporter plasmid (SLUG-pro-LUC#1) and treated with or without OSM (20 ng/mL). After 24 hours, the luciferase activities were measured and the results were presented as a relative percentage of light units (RLU) to shSC cells without OSM treatment. An asterisk (*) indicated the statistically significant differences between experimental group and shSC without OSM; a hash mark (#) indicated the statistically significant differences between experimental group and shSC with OSM. **B.** A549 cells were co-transfected with vector control (VC) or pcDNA3-STAT1 plasmid along with SLUG-pro-LUC#1 reporter plasmid, followed by treatment with or without OSM (20 ng/mL). An asterisk (*) indicated the statistically significant difference between experimental group and VC without OSM; a hash mark (#) indicated the statistically significant difference between experimental group and VC with OSM. **C.** A549 cells were transfected with different regions (−1121 to +186, −647 to +186, −414 to +186, −171 to +186) of SLUG promoter-driven luciferase-expressing reporter plasmids and treated with OSM (20 ng/mL). **D.** A549 cells were transfected with SLUG-pro-LUC#1 (GAS-WT) or a mutant sequence of putative GAS site (GAS-MUT), and treated with or without OSM (20 ng/mL). An asterisk (*) indicated the difference between experimental group and GAS-WT without OSM are statistically significant; A hash mark (#) indicated the difference between experimental group and GAS-WT with OSM are statistically significant. **E.** A549 cells were treated with OSM and stopped the reaction (by adding formaldehyde) at the indicated time. The association of SLUG promoter and STAT1 was examined by ChIP assay. Relative association, as reflected by the qPCR values, was calculated and normalized to the input DNA results. The qPCR primers were used to detect the specific sequence at −535 and −151 bp of SLUG promoter region. **F.** A549 cells were transfected with VC, pcDNA3-STAT1 plasmid (STAT1) or cells with shSC, shSTAT1-1 or restoration of STAT1 in shSTAT1-1 (shSTAT1-1+STAT1) were treated with OSM (20 ng/mL), and then subjected to the ChIP assay. The qPCR primers were used to detect the specific sequence at −535 bp of SLUG promoter region

Since SLUG mRNA levels were elevated in shSTAT1 cells and reduced in shSTAT3 cells, we further investigated whether STAT1 decreases SLUG expression through a transcriptional control. We constructed SLUG promoter-driven luciferase expression reporters (SLUG-pro-LUC#1) and test the effect of OSM and STAT1 on SLUG promoter activity. We observed that OSM inhibited SLUG promoter activity, while knockdown of STAT1 reversed the OSM-dependent inhibition resulted in a luciferase activity level similar to control cells (Figure 3A). On the other hand, overexpression of STAT1 augmented the OSM-dependent inhibition of SLUG promoter activity (Figure 3B). To map the potential STAT1 binding site on SLUG promoter region, we constructed luciferase reporter plasmids driven by different lengths of SLUG promoter (SLUG-pro-LUC#1 to 4, Figure 3C), and compared their transcriptional activities in the presence of OSM. We found a significant difference in luciferase activity between SLUG-pro-LUC#2 and SLUG-pro-LUC#3, meaning a potential OSM-dependent regulatory site between -647 and -414 of SLUG promoter, and suggested a potential STAT1 binding site within this region (Figure 3C). In support of this, we speculated a putative STAT1 binding sequence (gamma activated sequence (GAS), TTNNNNNAA) and, in line with our luciferase reporter results, found a potential site mapped at -599 bp upstream of SLUG transcriptional start site (Figure 3D). Moreover, mutagenesis (TTCGCGGAA to GGCGCGGCC) on this GAS sequence in SLUG-pro-LUC#1 released SLUG promoter activity from OSM-mediated suppression (Figure 3D). Nevertheless, through a chromatin immunoprecipitation (ChIP) assay with the anti-STAT1 antibody, we also showed that OSM induced STAT1 binding to SLUG promoter around this region (Figure 3E); the binding level (enrichment) of STAT1 on SLUG promoter was positively correlated with overexpressing or knockdown STAT1 (Figure 3F). These data indicated that OSM inhibited SLUG expression via inducing STAT1 binding to SLUG promoter to suppress its transcription.

### PIAS4 was involved in STAT1-mediated SLUG reduction

**Figure 4:**
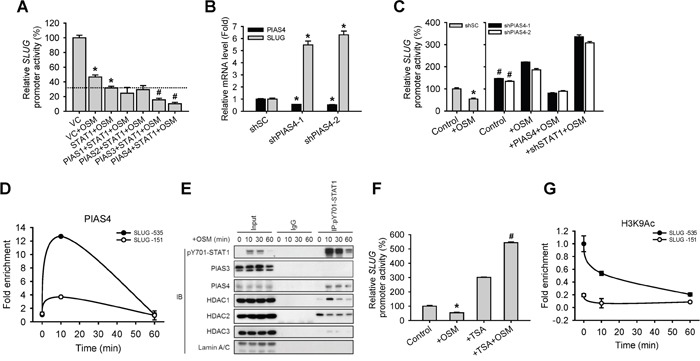
PIAS4 enhances the STAT1-mediated inhibition of SLUG expression **A.** A549 cells were co-transfected with vector control (VC) or pcDNA3-STAT1 plasmid, each subtype of PIAS-expressing plasmid and SLUG promoter (−1121 to +186)-driven luciferase-expressing reporter plasmid, and then treated with or without OSM (20 ng/mL). The SLUG promoter activity was analyzed with promoter assay. An asterisk (*) indicated that the difference between the experimental group and VC without OSM are statistically significant; A hash mark (#) indicated the difference between experimental group and STAT1 with OSM are statistically significant **B.** A549 cells with stable knockdown of PIAS4 (shPIAS4-1 and -2) were subjected to quantitative real-time PCR for analyzing the mRNA expression level of SLUG. **C.** A549 cells with stable knockdown of scramble shSC, shPIAS4-1 and -2, restoration of PIAS4 in shPIAS4 (+ PIAS4) and double knockdown of PIAS4 and STAT1 (+shSTAT1-1) were co-transfected with SLUG promoter (−1121 to +186)-driven luciferase-expressing reporter plasmid and treated with or without OSM (20 ng/mL). The SLUG promoter activity was analyzed with promoter assay. An asterisk (*) indicated the difference between experimental group and shSC without OSM are statistically significant; A hash mark (#) indicated the difference between experimental group and shSC with OSM are statistically significant. **D.** A549 cells were treated with OSM (20 ng/mL) and then subjected to the ChIP assay. **E.** Proteins which interacted with STAT1 were detected by co-immunoprecipitation using nuclear extracts from OSM-treated cells. Lysates were incubated with anti-STAT1 antibody or nonspecific immunoglobulin G (IgG), and bound proteins detected by Western blotting assay. **F.** A549 cells were co-transfected with SLUG promoter (−1121 to +186)-driven luciferase-expressing reporter plasmid and treated with OSM (20 ng/mL), TSA alone or TSA combined with OSM. The SLUG promoter activity was analyzed with promoter assay. An asterisk (*) indicated the difference between experimental group and Control are statistically significant; A hash mark (#) indicated the difference between experimental group and cells with OSM are statistically significant. **G.** A549 cells were treated with OSM (20 ng/mL) in 10 and 60 minutes, and then subjected to the ChIP assay to detect H3K9 acetylation on SLUG promoter region.

To dissect how a transcription factor like STAT1 mediates transcriptional suppression, we speculated certain co-factors may be required for this regulatory pathway. According to Shuai and Liu's review [15], Protein Inhibitor of Activated STAT (PIAS) proteins may play as a co-factor of STATs-dependent transcriptional control. We firstly generated stable cell lines expressing 4 different PIAS proteins (PIAS1 to 4). SLUG promoter reporter assay and qPCR in the presence of OSM revealed that though the 4 PIAS proteins showed suppressive effect on SLUG promoter activity as well as mRNA expression in cells when co-expressed with STAT1, PIAS3 and PIAS4 demonstrated the most statistically significant effect with STAT1 on suppressing SLUG (Figure 4A and Supplementary Figure S1G). Previous studies showed that PIAS4 could interact with STAT1 while PIAS3 interact with STAT3 [15]. To study whether PIAS4 participate in STAT1-mediated SLUG transcriptional suppression, we knocked down PIAS4 (shPIAS4) in A549 cells and showed that shPIAS4 cells had increased SLUG mRNA level (Figure 4B) and SLUG promoter activity than control cells, while OSM treatment augments this difference (Figure 4C). By ChIP assay with the anti-PIAS4 antibody, we showed PIAS4 binds to the SLUG promoter region (Figure 4D) where STAT1 binding (Figure 3E). These results indicated that PIAS4 may co-operate with activated STAT1 and caused the reduction of SLUG at the transcription level.

HDAC has been reported to participate in PIAS-mediated transcriptional regulation [16, 17]. We then tested if epigenetic events were elicited during the co-operation of STAT1 and PIAS4 for transcriptional suppression of SLUG. First, a co-immunoprecipitation assay results showed that HDAC1 and PIAS4 bind to phosphorylated STAT1 in nucleus 10 minutes after OSM treatment (Figure 4E). In addition, pre-treating cells with HDAC inhibitor, Trichostatin A (TSA), reversed the OSM-induced inhibition of SLUG promoter activity (Figure 4F). Because we found that HDAC1 interacted with STAT1 and PIAS4, we wondered any histone modification on the SLUG promoter region upon OSM treatment. We detected the histone3 lysine9 acetylation (H3K9Ac), which is a gene-activated marker, at SLUG promoter. The level of H3K9Ac was decreased in one hour after OSM administration (Figure 4G). These data indicated that the OSM-dependent inhibition of SLUG and elevation of MET signaling was mediated by a STAT1-PIAS4-HDAC1-dependent pathway through an epigenetically transcriptional control.

### PIAS3 blocked the binding of STAT3 on SLUG promoter

**Figure 5:**
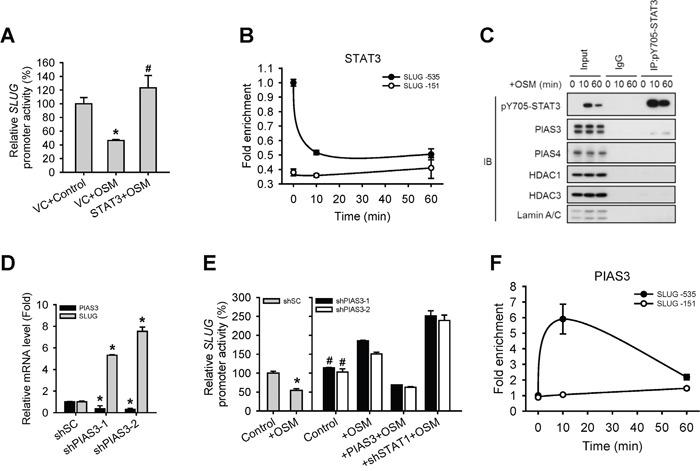
PIAS3 suppressed the STAT3-mediated expression of SLUG **A.** A549 cells were co-transfected with vector control (VC) or pcDNA3-STAT3 plasmid and SLUG promoter (−1121 to +186)-driven luciferase-expressing reporter plasmid, and then treated with or without OSM (20 ng/mL). The SLUG promoter activity was analyzed with promoter assay. An asterisk (*) indicated the difference between experimental group and VC control are statistically significant; A hash mark (#) indicated the difference between experimental group and VC with OSM are statistically significant. **B.** A549 cells were treated with OSM at 10 and 60 minutes and then subjected to the ChIP assay to detect STAT3 on SLUG promoter. **C.** Proteins which interacted with STAT3 were detected by co-immunoprecipitation using nuclear extracts from OSM-treated cells. Lysates were incubated with anti-STAT3 antibody or nonspecific immunoglobulin G (IgG), and bound proteins detected by Western blotting assay. **D.** A549 cells with stable knockdown of PIAS3 (shPIAS3-1 and 2) were subjected to qPCR for analyzing the mRNA expression level of SLUG. **E.** A549 cells with stable knockdown of scramble control (shSC), PIAS3 (shPIAS3-1 and -2), restoration of PIAS3 in shPIAS3 (+ PIAS3) and double knockdown of PIAS3 and STAT1 (+shSTAT1-1) were co-transfected with SLUG promoter (−1121 to +186)-driven luciferase-expressing reporter plasmid and treated with or without OSM (20 ng/mL). The SLUG promoter activity was analyzed with promoter assay. An asterisk (*) indicated the difference between experimental group and shSC without OSM are statistically significant; A hash mark (#) indicated the difference between experimental group and shSC with OSM are statistically significant. **F.** A549 cells were treated with OSM at the indicated time and then subjected to the ChIP assay to detect PIAS3 on SLUG promoter.

There is evidence to suggest that abnormal STAT3 signaling promotes progression of human cancers by either inhibiting apoptosis or inducing cell proliferation, angiogenesis and metastasis [18]. However, STAT3 seemed to have little effect on OSM-dependent regulation of SLUG expression in our system (Figure 2), even though it was phosphorylated and translocated to the nucleus (Figure 1). Thus, we wonder why cells choose to response to the suppressive effect of STAT1 rather than the activation effect of STAT3 in terms of SLUG expression, cell motility, and tumor metastasis, while OSM activates both. To understand the role of OSM-activated STAT3 in LAC cells, we overexpressed STAT3 in A549 cells before OSM treatment. The SLUG promoter assay showed that STAT3 recovered the inhibited SLUG promoter activity mediated by OSM (Figure 5A). Moreover, we showed with ChIP assay that STAT3 may bind to the similar region in SLUG promoter, and the enrichment binding level of STAT3 on SLUG promoter is significantly decreased upon OSM treatment (Figure 5B). The fact that OSM encouraged the dissociation of phosphorylated STAT3 from SLUG promoter triggered us to hypothesize that other binding partner(s) may involve. There has been evidence showed that PIAS3 repress the expression of the downstream gene by blocking the binding of STAT3 to a promoter [19, 20]. Interestingly, co-IP results in our system also showed the interaction between STAT3 and PIAS3 but not PIAS4 (Figure 5C). To understand the role of PIAS3 in OSM-mediated SLUG regulation in LAC cells, we knocked down PIAS3 (shPIAS3) in A549 cells. We found that shPIAS3 cells had increased SLUG mRNA level as well as SLUG promoter activity in comparison to shSC control cells, while OSM treatment augmented this difference (Figure 5D and 5E). By ChIP assay, we showed increased binding of PIAS3 to SLUG promoter region 10 minutes after OSM treatment but then decreased afterward (Figure 5F). These data revealed that STAT3 is able to maintain the SLUG expression. After OSM treatment, PIAS3 bound to activated STAT3 and blocked binding of STAT3 on SLUG promoter region.

In summary, we showed that both STAT1 and STAT3 were phosphorylated on tyrosine residue of c-terminal of STAT proteins, and translocated to the nucleus after OSM treatment. A knockdown study indicated that STAT1 and STAT3 oppositely regulated SLUG expression at its transcription level, as well as cell morphology and motility. Using reporter assay, co-immunoprecipitation (co-IP), and chromatin immunoprecipitation (ChIP) assays, we showed that PIAS4 and PIAS3 respectively bind to STAT1 and STAT3, leading to enhanced STAT1 binding to SLUG promoter and alleviating STAT3 from SLUG promoter, causing an epigenetic change and silenced SLUG promoter.

## DISCUSSION

Tumor development with metastasis is the leading causes of mortality in lung cancer patients, and EMT has been considered a critical mechanism regulating the metastatic progression of cancer [21]. To improve effective strategies for the prediction, diagnosis and treatment of metastasis of lung cancer, the molecular mechanisms controlling metastasis must be characterized. Targeting the EMT pathway or enhancing MET has been suggested as a promising therapeutic method to improve patient survival [22]. Here, we documented OSM inhibits the migration, invasion and proliferation of lung adenocarcinoma cell lines through a STAT1-dependent mechanism. Moreover, the EMT regulator SLUG was decreased and the MET marker E-cadherin was elevated by OSM, resulting in reduced motility, proliferation and metastasis in LAC cell lines. Our findings suggested that OSM or its downstream target may have a therapeutic potential for lung cancer treatment and its molecular mechanism needs further investigation.

We previously found that OSM suppresses cell motility and induces MET process in LAC cells through inhibiting SLUG expression. However, there is an absence of work studying the mechanistic role of OSM in moderating lung EMT-MET and metastatic process. In our data, we found that OSM exerts its suppressive effect on cell motility via inducing STAT1 activation, leading to increased E-cadherin and suppressed SLUG expression. Experimental metastasis assay in the mouse model showed OSM significantly suppresses the formation of metastatic pulmonary nodules, suggesting OSM is an effective and potent factor in the reduction of EMT and metastasis in LAC cells.

**Figure 6:**
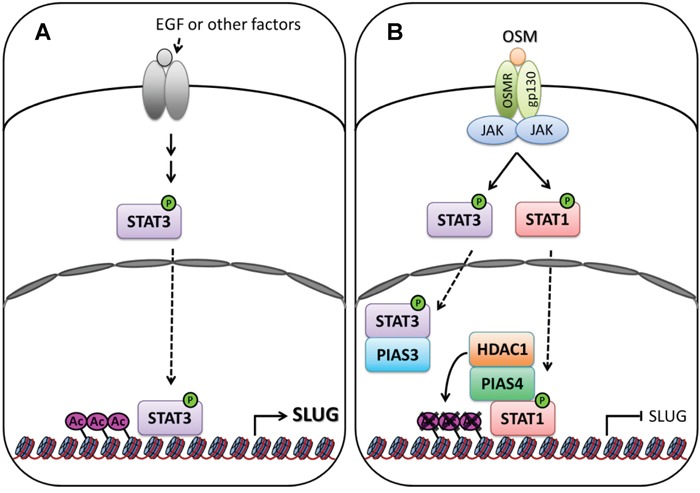
Signaling of OSM decreases SLUG expression in LAC **A.** In tumor microenvironment, autocrine or paracrine factors, including EGF and IL-6, from surrounded stromal or immune cells activate intrinsic growth factor- or cytokine- associated receptors tyrosine kinases, and downstream kinases which, in turn, phosphorylate STAT3 [28, 29]. Phosphorylated STAT3 translocate to nucleus and bind on SLUG promoter to maintain the expression of SLUG gene. **B.** OSM activates STAT1 and STAT3 via binding to OSM receptor (OSMR) and gp130 complex. The phosphorylated STAT1 translocate to the nucleus, bind to PIAS4 and HDAC1, and bind at SLUG promoter region. Acetylation of Histone 3 Lysine 9 (H3K9Ac) near this region was removed by HDAC1, resulting in silence the expression of SLUG gene. Phosphorylated STAT3 translocate to the nucleus, but it was blocked the binding on SLUG promoter region by PIAS3.

The STAT proteins are well-studied transcription factors that receive a variety of regulatory signals from cell-surface receptors and modulate downstream gene expression. Dysregulation of STAT function contributes to numerous human diseases including cancer. Previous reports showed that STAT3 promotes tumor cell survival, proliferation, motility and immune tolerance and is considered as an oncogene [23]. In contrast, STAT1 induces the anti-proliferative and pro-apoptotic responses in tumor cells, enhances inflammation and innate and adaptive immunity. Moreover, STAT1-null mice increase susceptibility to tumors [13]. We found STAT1 is a negative regulator of the SLUG expression through a transcriptional control. However, the fact that OSM induces the phosphorylation and activation of both STAT1 and STAT3 and yet still presented anti-tumor effects poses a paradox why LAC cells selectively response to activated STAT1 and ignore the pro-oncogenic STAT3. Moreover, previous studies on STAT1 and STAT3 focused on individual signaling pathway with little information about their coordination and mutual regulation to each other. In our findings, though OSM activated STAT1 and STAT3 simultaneously, STAT1 showed its dominant effect on control of SLUG expression due to the involvement of PIAS3 and PAIS4 proteins (Figure 6). PIAS3 blocked STAT3 binding on the promoter of SLUG to suppress SLUG transcription, whereas PIAS4 bound to phosphorylated STAT1 and HDAC1 on SLUG promoter to silence the gene expression. With the participation of PIAS3 and PIAS4, STAT1 dominates OSM effect on SLUG gene transcription.

The functional effects of OSM in different cell models have been reported controversial. Studies on melanoma, osteosarcoma, neuroblastoma cancer cells demonstrated the anti-proliferative effect of OSM [11, 24], while studies on ovarian cancer and Ewing sarcoma cells showed pro-proliferative effect [25, 26]. These reports suggest that different cellular content of cancer cells may result in the divergent net outcome of OSM. The contradiction in different cell types probably is the reason that prevents OSM from the clinical trial after OSM has been patented as an anti-proliferation drug (US Patent # 5428012). We found that manipulating PIAS3 and PIAS4 can change the dominancy of STAT1 and STAT3 in response to OSM, and lead to different regulation of SLUG expression. This may shed some light on the potential cause of divergent cellular responses to OSM in different cell types. Conclusively, our mechanistic investigation of OSM-dependent tumor suppressive effect not only revealed the involvement of STAT1 to suppress SLUG expression and cellular motility but also provided insight how dominancy of STATs is switched.

## MATERIALS AND METHODS

### Reagents

Recombinant human OSM was purchased from PeproTech. Trichostatin A (TSA) was purchased from Sigma-Aldrich. Matrigel was purchased from BD Biosciences.

### Cancer cell lines and culture conditions

A549 lung adenocarcinoma cell line was obtained from the American Type Culture Collection in 2012 and tested positive for human origin. The CL1-5 lung adenocarcinoma cell line was established previously [27]. All cell lines were maintained in RPMI-1640 medium (Sigma-Aldrich) supplemented with 10% fetal bovine serum and Penicillin-Streptomycin (Gibco, Invitrogen) in a humidified incubator with 5% CO_2_ at 37°C.

### Immunofluorescent confocal microscopy

Cells were fixed with 4 % paraformaldehyde and permeabilized with 0.2% Triton X-100 in phosphate saline buffer (PBS) for 10 minutes each, followed by blocking in 5% bovine serum albumin for 1 hour at room temperature. Cells were hybridized individually with the indicated primary antibodies (in Supplementary Table S1) overnight at 4°C, followed by fluorescence-labeled secondary antibodies. Stained cells were mounted by glass coverslips using the mounting medium containing DAPI (Vector Lab) and examined under a confocal microscope (FV10i, Olympus).

### Western blotting assay

Western blotting was performed according to the recommended protocol from Cell Signaling Technology. The aliquot protein sample was mixed with Laemmli sample buffer and boiled at 100°C for 5 min and separated on 10% SDS-PAGE. The proteins were transferred to PVDF membrane (Pall Corporation). Primary and secondary antibodies were added as indicated (in Supplementary Table S1). Quantification of protein was detected by the Luminata Western HRP substrate detection system (Millipore).

### Animals and experimental metastatic assay

All procedures involving animals were performed in accordance with the institutional animal welfare guidelines of Taipei Veterans General Hospital. CL1-5 cells were harvested, washed, resuspended in PBS (in a total volume of 100 mL) were injected into the tail vein of 8-week-old male BALB/c nude mice (BioLasco Taiwan Co.) at 1×10^6^ cells/injection.

### Migration and invasion assay

A FluoroBlok 24-Multiwell Insert System with an 8-μm pore size polyethylene terephthalate membrane (BD Falcon) was used to test cell motility. Each well was filled with 700 μL medium, and cell suspensions were seeded into the insert chamber at a density of 2.5×10^4^ cells in 300 μL medium. After 24 hours, the medium was removed, and the chamber was washed with PBS and fixed in 100% methanol overnight at −20°C. The reverse side of the membrane facing the lower chamber was stained with propidium iodide (Sigma-Aldrich) for 30 minutes, and the migratory cells were then visualized under an inverted fluorescent microscope. Cell number was quantitated using ImageJ software. For the invasion assay, the membrane was coated with Matrigel (BD Biosciences) diluted with an equal volume of serum-free medium and incubated for at least 1 hour at 37°C before the cells were seeded.

### Real-time PCR (qPCR)

RNA was extracted from cells using TriPure isolation reagent (Roche Life Science) according to the manufacturer's protocol. Extracted total RNA was reverse transcribed into cDNA using the random hexamer primer with Transcriptor First Strand cDNA Synthesis Kit (Roche Life Science). Each cDNA was equally diluted for subsequent PCR amplification with the KAPA SYBR FAST ABI Prism 2X qPCR Master Mix (KAPA Biosystems) using StepOnePlus Real-Time PCR System (Applied Biosystems). The sequence of the primers designed to detect specific genes is available in Supplementary Table S2. The relative gene expression normalized to 18S was calculated using the 2−ΔΔCt methods.

### Proliferation assay

Cells were seeded into 96-well cell culture plates at a density of 1×10^3^ cells/well in 100 μL media and allowed to adhere overnight. The media was aspirated and replaced with fresh complete medium with or without recombinant OSM as described. An alamarBlue Cell Viability Assay (Thermo Fisher Scientific) was carried out according to the manufacturer's protocol to assess the changes in relative cell density every 24 hours.

### Luciferase reporter assay

A549 or CL1-5 cells were grown to approximately 60-70% confluence in 24-well plates and transfected with pGL3-basic plasmid containing the various region of SLUG promoter or a mutant sequence of putative GAS site. Transfected cells were incubated over one night and treated with or without OSM. After 24 hours of treatment, luciferase activity was analyzed with the Dual-Luciferase Reporter Assay (Promega) according to the manufacturer's protocol.

### Preparation of nuclear and cytosolic extracts and co-immunoprecipitation

Nuclear and cytosolic extracts were isolated with a NE-PER Nuclear and Cytoplasmic Extraction Reagents (Thermo Fisher Scientific). The nuclear and cytosolic extracts were used in Western blotting or co-immunoprecipitation assays later. In the co-immunoprecipitation assay, protein G Dynabeads (Invitrogen) was incubated with 2.5 μL antibody for 4 hours at 4°C. Next, the cell extracts 350 μg were incubated with antibody-conjugated beads overnight at 4°C. The beads were separated by magnetic base and wash 3 times (50 mM Tris pH 7.5, 170mM NaCl, 13mM MgCl_2_, 0.5% NP40, 0.3% Triton X-100 and protease inhibitor cocktail). Finally, the beads were added 25 μL 1× SDS sample buffer and heat for 10 min at 90°C to elute the protein. All samples were analyzed by subsequent Western blotting.

### Chromatin immunoprecipitation (ChIP) assay

The chromatin immunoprecipitation assay was performed according to the protocol for the LowCell# ChIP kit (Diagenode) and quantified by real-time PCR. Antibodies and sequence of primer sets to detect specific promoters are listed in Supplementary Table S2.

### Short hairpin RNA

RNAi reagents were obtained from the National RNAi Core Facility located at the Institute of Molecular Biology/Genomic Research Center, Academia Sinica, supported by the National Core Facility Program for Biotechnology Grants of NSC (NSC 100-2319-B-001-002). Target sequences of shRNA are listed in Supplementary Table S2.

### Statistical analysis

The results are reported as the mean±SD. Statistical analyses were carried out using Student t-test. A *p*-value <0.05, as denoted with “*” or “#” in figures, was considered statistically significant.

## SUPPLEMENTARY FIGURE AND TABLES


