# Supplementary material for: Oncostatin M suppresses metastasis of lung adenocarcinoma by inhibiting SLUG expression through coordination of STATs and PIASs signalings
Source: Oncotarget. 2016 Jul 29;7(37):60395–406. doi: 10.18632/oncotarget.10939 (PMC5312391; doi:10.18632/oncotarget.10939)
Supplement: Supplementary file 1 [file oncotarget-07-60395-s001.pdf]

Oncostatin M suppresses metastasis of lung adenocarcinoma by inhibiting SLUG expression through coordination of STATs and PIASs signalings

SUPPLEMENTARY FIGURE AND TABLES

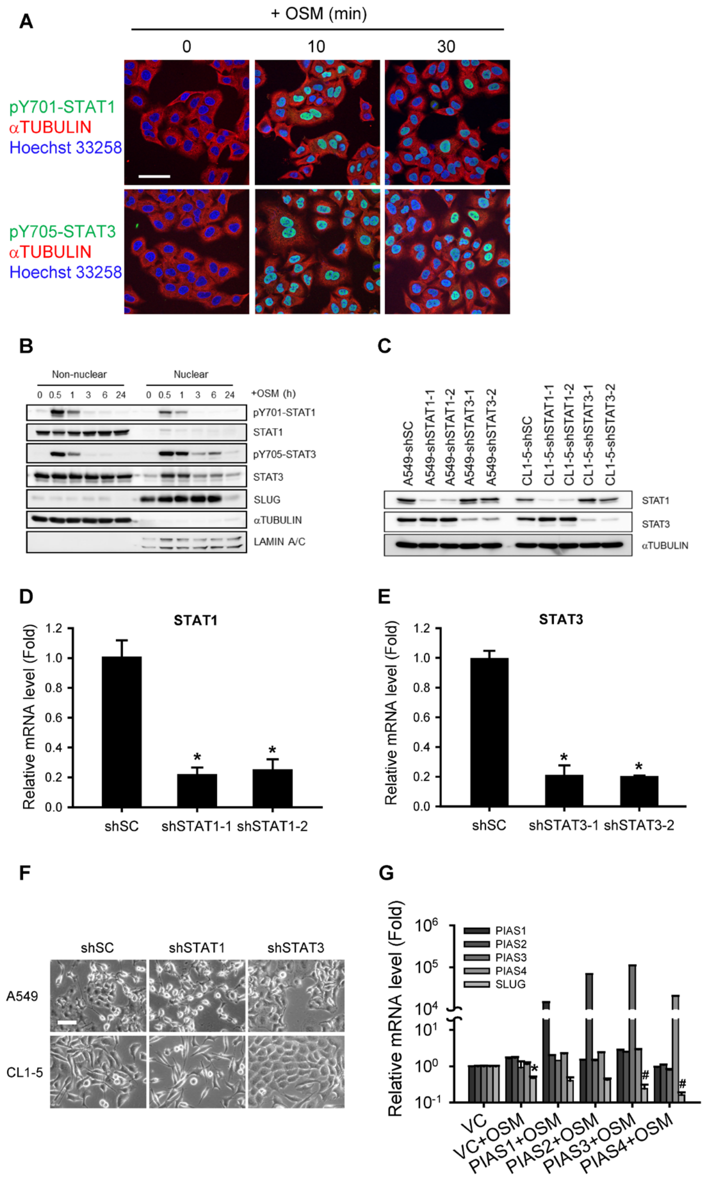

Supplementary Figure S1: A. A549 cells were treated with 20 ng/mL OSM for the indicated durations were subjected to immunofluorescent staining of pY701-STAT1 (green), pY705-STAT3 (green), αTUBULIN (red) and Hoechst 33258 (blue), and observed by confocal microscope. pY701-STAT1 and pY705-STAT3 indicated the phosphorylated STAT1 and STAT3, respectively. Scale bar = 50 µm. **B.** CL1-5 cells were treated with 20 ng/mL OSM for the indicated durations. Cell lysates were collected and use NE-PER Kit to extract the nuclear and cytoplasmic protein fractions. The protein extracts were subjected to Western blotting assay. (C to E) A549 and CL1-5 cells were stably knocked down STAT1 (shSTAT1-1 and -2) and STAT3 (shSTAT3-1 and -2) as well as cells with scrambled shRNA control (shSC), then subjected to a Western blotting (C) or a real-time PCR (D and E) for analysis of STAT1 or STAT3 protein and mRNA level, respectively. **F.** The morphology of A549 and CL1-5 cells with shSC, shSTAT1, and shSTAT3 were observed under optical microscope. Scale bar = 50 µm. **G.** A549 cells were transfected with vector control (VC) or PIAS1 to 4-expressing plasmid, and then treated without or with OSM (20 ng/mL). The PIAS and SLUG mRNA level were analyzed by quantitative real-time PCR. An asterisk (*) indicated the difference between experimental group and VC without OSM are statistically significant; A hash mark (#) indicated the difference between experimental group and VC with OSM are statistically significant.

Supplementary Table S1: List of antibodies used in the report

Target	Supplier	Catalog number	Appilication
STAT1	Cell Signaling	9175	IF, WB
STAT3	Cell Signaling	12640	IF, WB, ChIP
phospho-Y701-STAT1	Cell Signaling	9167	WB
phospho-Y705-STAT3	Cell Signaling	9145	WB
PIAS3	Cell Signaling	9042	WB, ChIP
PIAS4	Cell Signaling	4392	WB, ChIP
SLUG	Cell Signaling	9585	WB
HDAC1	Cell Signaling	5356	WB
HDAC2	Cell Signaling	5113	WB
HDAC3	Santa Cruz	sc-11417	WB
E-cadherin	BD Biosciences	610181	WB
α-Tubulin	Sigma-Aldrich	T6074	IF, WB
Lamin A/C	Cell Signaling	2032	WB
STAT1	Santa Cruz	sc-346X	ChIP
H3K9Ac	GeneTex	GTX61103	ChIP
GAPDH	Cell Signaling	5174	WB
Dylight549-labeled anti-mouse IgG	Rockland	610-742-124	IF
Dylight488-labeled anti-rabbit IgG	Biolegend	406404	IF
HRP-conjugated anti-rabbit IgG	Genetex	225856	IP-WB
HRP-conjugated anti-mouse IgG	Genetex	225857	IP-WB

Supplementary Table S2: List of primer and shRNA sequences used in the report

Target	sequence	application
STAT1	Forward: ACTTTCCCTGACATCATTCGCA<br>Reverse: GAGCAGGTTGTCTGTGGTCTG	Real-time PCR
STAT3	Forward: AGACCCAGATCCAGTCCGTG<br>Reverse: TTGGTCTTCAGGTATGGGGCA	Real-time PCR
PIAS3	Forward: CGGAGCTGGGCGAATTAAAG<br>Reverse: TTCCGTCCACTCTTGTTCCG	Real-time PCR
PIAS4	Forward: TAAACGGACTGGGACGGTTG<br>Reverse: TCAACTCCACCTGTCTTGGC	Real-time PCR
SLUG-535	Forward: CAGAGTCCCAGGAGAGCGTC<br>Reverse: GGCATCTGGAGAGGTTTGCC	ChIP-qPCR
SLUG-151	Forward: AAAACAGCCCATTTTGAACCA<br>Reverse: GATTTCAGCTCCTCCCTCTGG	ChIP-qPCR
STAT1 (shSTAT1-1)	CTGGAAGATTTACAAGATGAA	shRNA
STAT1 (shSTAT1-2)	CTGGAAGATTTACAAGATGAA	shRNA
STAT3 (shSTAT3-1)	GCAAAGAATCACATGCCACTT	shRNA
STAT3 (shSTAT3-2)	GCACAATCTACGAAGAATCAA	shRNA
PIAS3 (shPIAS3-1)	CCGACATCCAAGGTTTAGATT	shRNA
PIAS3 (shPIAS3-2)	ATCTGAGTTCGGACGGAATTA	shRNA
PIAS4 (shPIAS4-1)	CACCGAATTAGTCCCACAGAA	shRNA
PIAS4 (shPIAS4-2)	GCTCTACGGAAAGTACTTAAA	shRNA
